# Supplementary material for: The netrin receptor UNC-40/DCC assembles a postsynaptic scaffold and sets the synaptic content of GABAA receptors
Source: Nat Commun. 2020 May 29;11:2674. doi: 10.1038/s41467-020-16473-5 (PMC7260190; doi:10.1038/s41467-020-16473-5)

**Supplementary Information**

# The netrin receptor UNC-40/DCC assembles a postsynaptic scaffold and sets the synaptic content of $GABA_A$ receptors

Zhou et al.

# Supplementary figures

## Supplementary figure 1

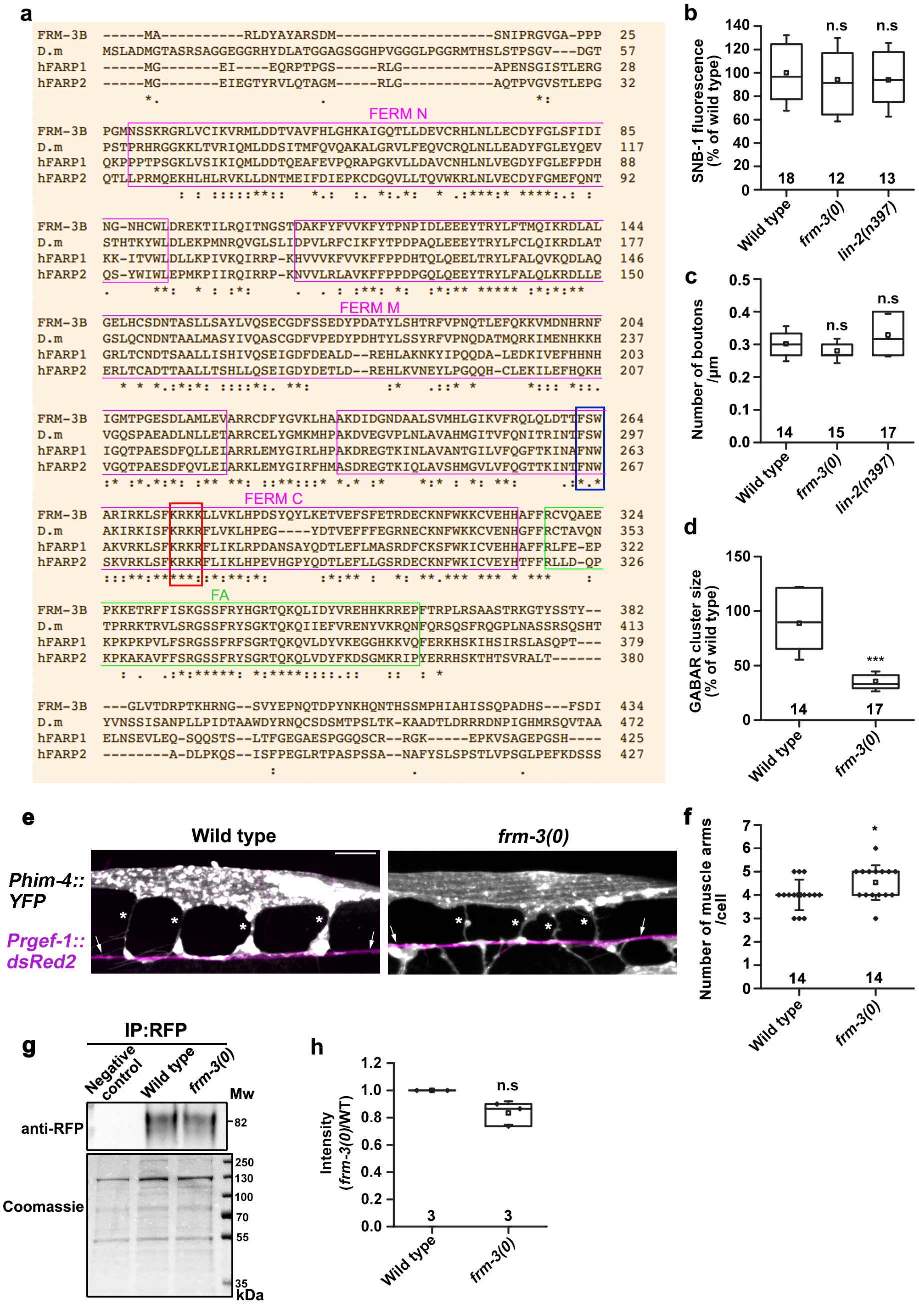

**Supplementary figure 1. GABA boutons and muscle arms are not modified in *frm-3* and *lin-2* mutants.**

(a) Protein alignment of *C. elegans* FRM-3 with its Drosophila and human orthologs. Amino acid sequences of FERM-FA domains of *C. elegans* FRM-3 (common to FRM-3A and FRM-3B isoforms), *Drosophila melanogaster* FARP (D.m, UniProt entry: A0A0C4DHA1), human FARP1 (UniProt entry: Q9Y4F1), human FARP2 (UniProt entry: O94887) were aligned using CLUSTAL omega software. "KRKK" residues (red box) defines a critical phospholipid binding site[1] and "FxW" residues (blue box) support FERM+FA self-oligomerization[2]. Three FERM subdomains were labeled by magenta box. FA (FERM adjacent) domain was shown in green box.

(b) Fluorescence intensity of SNB-1-GFP expressed in GABA motoneurons of wild-type, *frm-3(gk585)* and *lin-2(n397)* animals. Data are normalized to the wild type. Box plot as in figure 1e. One-way ANOVA followed by Tukey's multiple comparison tests of each group compared to the wild type. n.s: not significant.

(c) Number of GABAergic boutons in wild-type, *frm-3(gk585)* and *lin-2(n397)* animals. Box plot as in figure 1e. One-way ANOVA followed by Tukey's multiple comparison tests of each group compared to the wild type. n.s: not significant.

(d) Size of the GABAR clusters in the wild type and *frm-3(gk585)* mutants. Data are normalized to the wild type. Box plot as in figure 1e. Two-tailed Student's t test, $^{***}p<0.001$.

(e) Transgenic *trIs25* allele animals expressing YFP in distal muscle cells and dsRed2 in motoneurons were observed in wild-type and *frm-3(0)* mutant backgrounds.

(f) The number of muscle arms (indicated with stars) per muscle cell was counted and compared by two-tailed Student's t test. n.s, not significant. Box plot as in figure 1g.

(g) The $GABA_AR$ overall level is normal in *frm-3(gk585)* mutants. Lysates of RFP-$GABA_AR$ (knock-in *kr296[rfp-UNC-49])* animals were immunoprecipitated with anti-RFP Trap-A beads, in the *frm-3(gk585)* (*frm-3(0)*) and in the wild-type background (wild type). The immunoprecipitated samples were immunoblotted with an anti-RFP antibody. The N2 Bristol strain was used as a negative control. Mw: molecular weight marker. The molecular weight of the RFP-$GABA_AR$ band is indicated. This experiment was repeated 3 times.

(h) The intensity of RFP-$GABA_AR$ immunoprecipitated bands was assessed and normalized to the intensity of bands revealed by Coomassie staining, in the *frm-3(gk585)* and in the wild-type background (wild type). Data are presented as a ratio of normalized intensity to the wild type. Box plot as in figure 1g. Two-tailed Student's t test, n.s: not significant.

Scale bar = 10 µm.

**Supplementary figure 2**

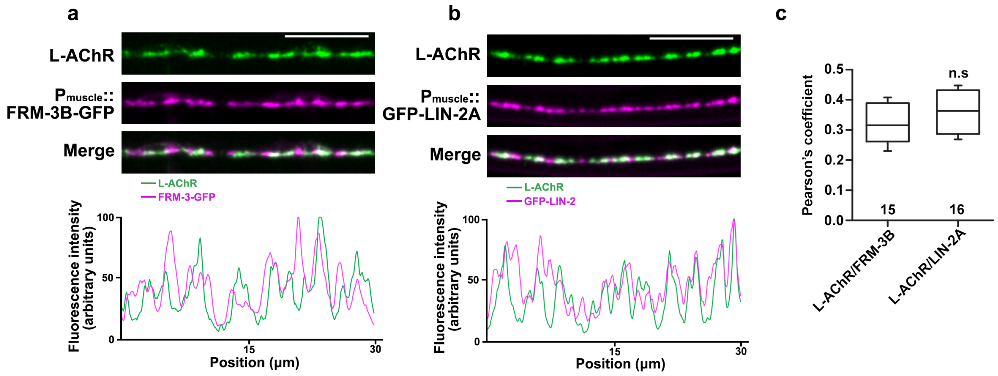


**Supplementary figure 2. FRM-3 and LIN-2 are present at excitatory cholinergic synapses.**

(a) Confocal detection of the L-AChR UNC-29-RFP subunit expressed in the *unc-29(kr208::rfp)* knock-in strain and FRM-3B-GFP specifically expressed in muscle. The fluorescence profiles indicate UNC-29-RFP and FRM-3-GFP fluorescence intensities along the nerve cord from the pictures above.

(b) Confocal detection of L-AChRs as in (a) and GFP-LIN-2A specifically expressed in muscle. The fluorescence profiles indicate UNC-29-RFP and GFP-LIN-2A fluorescence intensities along the nerve cord from the pictures above.

(c) Pearson's correlation coefficient between L-AChR and FRM-3B or LIN-2A. Box plot as in figure 2d. Mann Whitney's test, ns: not significant.

Scale bars = 10 µm.

## Supplementary figure 3

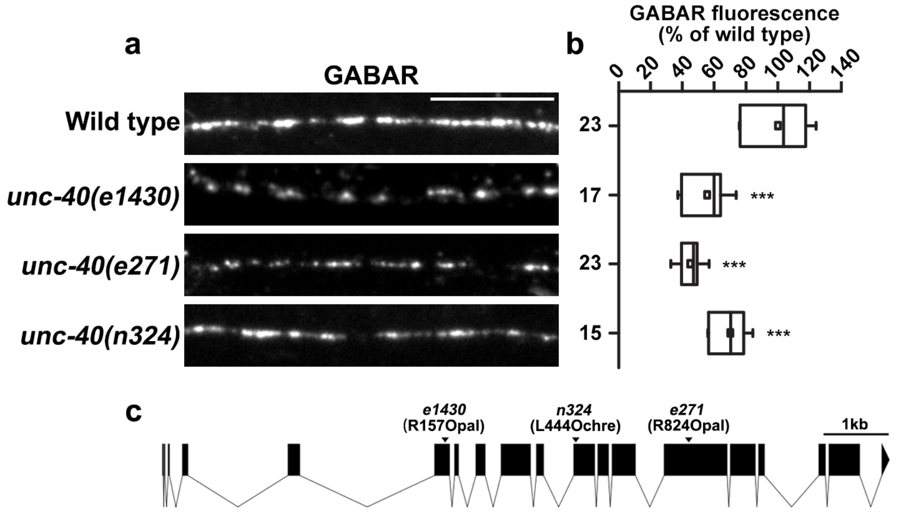


**Supplementary figure 3. The content of GABAR at synapses is decreased in three different *unc-40* mutant alleles.**

(a) Spinning disk images of RFP-GABAR in the wild type and in *unc-40(e1430)*, *unc-40(e271)* and *unc-40(n324)* mutants.

(b) GABAR fluorescence levels were calculated for each group and normalized to the wild type. Box plot as in figure 1e. One-way ANOVA followed by Turkey's multiple comparison tests. $^{***}p<0.001$.

(c) Genomic locus of *unc-40* showing the different mutant alleles used in this study.

Scale bar = 10 µm.

**Supplementary figure 4**

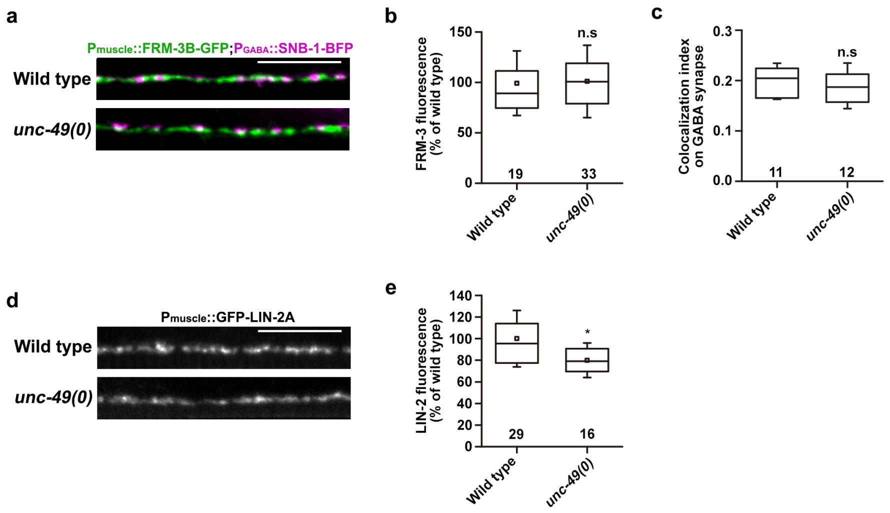


**Supplementary figure 4. GABA$_A$Rs do not control synaptic localization of FRM-3 and LIN-2.**

(a) Confocal detection of FRM-3B-GFP expressed in muscle cells and of GABAergic boutons labeled by SNB-1-BFP in wild-type or *unc-49(e407)* animals.

(b) The fluorescence intensity of FRM-3B-GFP was quantified and normalized to the wild type. Box plot as in figure 1e. One-way ANOVA followed by Tukey's multiple comparison test, n.s not significant.

(c) Mander's overlap correlation indicates the percentage of FRM-3-GFP signal that overlaps with SNB-1-BFP signal. Box plot as in figure 2d. Kruskal-Wallis test followed by Dunn's post test, n.s not significant.

(d) Confocal detection of GFP-LIN-2 expressed in muscle cells of the wild type or *unc-49(e407)* mutant animals.

(e) The fluorescence intensity of GFP-LIN-2A was quantified and normalized to the wild type. Box plot as in figure 1e. One-way ANOVA followed by Tukey's multiple comparison test, *p<0.05, n.s not significant.

Scale bars = 10 µm.

## Supplementary figure 5

**a**

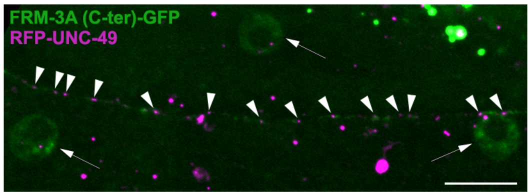


**b**

### cNLS Mapper Result

**Predicted NLSs in query sequence**

```
TYSSTYGLVTDRPTKHRNGSVYEPNQTDPYNKHQNTHSSMPHIAHISSQP  50
ADHSFSGTLDARVGKSVTRDPSTSVTQHRLRQLKRSERCISDVDTVERPQ  100
RSSCRPVSHAINISSTSSKEQPDLSVSLPNVLSDDLQMVCKEIEIEQNDP  150
PKSVSGDNFQQRRSSRDYDNVSEDSYRLSDHERSTRSEVGVGSKFAAATI  200
FNSTFVARKPGSNVVKRVVAHTKSTPNSTDDEEGALKSASEYQTFRHIKE  250
YPFARNANIVPIEIDGPNVDLSARRSPAATTTTTRTYANTTTTVTTTSKV  300
LTSSGAVLMKPKVISNDHEPSSGTHSSRVSPESGTYGALGPLPGRVITKE  350
NMMITPEGFKEKKAKPNPPPKPSFPVQAVHSETEVVREIKTERASSHPLV  400
HMQEEIPFSRAPLKFEDKKERKSSLSRPALISVQSEDNPDVQKCHLFNSD  450
IPYTLTMRNVENTQSLPYSSFKDVSTAKKQGYESNSLKRVSKSPEFKRRK  500
SLDLVPRKRLPSPGNFSAQDHTISPTTPDSDVLEYLLRRRSLLGDKSVIK  550
TKSKRTDPRRQTQPVRFDLPPSPCSPTAGGSTPFISILNDDLFDECVSES  600
RSLHEDMDRLDKTTPHNQSISSFMSQKSRRDSEDSDVMPPPPEEILNKAM  650
ISSSRPAPPPPPPPKSLAAAVKVAEMKAAAAGKIYYSGSSRTGKITDSPPF  700
IDDSPKNSSASDAPSVPSPQKNCFSDF                         727
```

**Supplementary figure 5. Mislocalization of FRM-3 C-terminus domain in muscle cells.**

(a) The C-terminus of FRM-3A (413-1091 aa) fused to GFP showed weak colocalization with GABAR (RFP-UNC-49, triangles) and ectopic localization in the nucleus (arrows). Note that the C-terminus of FRM-3A is the only fusion protein analyzed in this study to show nuclear localization. Scale bar=10 μm.

(b) The prediction of nuclear localization signal in the C-terminus of FRM-3A was achieved using the cNLS mapper server, which predicts importin α-dependent nuclear localization signals (Highlight in red). This program is based on (Kosugi S. et al., 2009)[3].

http://nls-mapper.iab.keio.ac.jp/cgi-bin/NLS_Mapper_form.cgi

## Supplementary figure 6

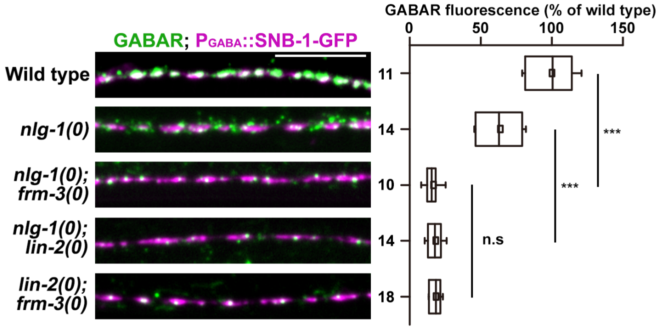


**Supplementary figure 6. NLG-1 requires FRM-3 and LIN-2 to selectively target GABA$_A$R.**

Confocal detection of RFP-labeled GABA$_A$R expressed in the *unc-49::rfp* knock-in allele *kr296* in wild-type animals and *nlg-1(ok259)* mutant, alone or in combination with *frm-3(gk585), lin-2(n397),* and in the double mutants *lin-2(n397); frm-3(gk585)*. GABAergic boutons are labeled with SNB-1-GFP. GABAR fluorescence level of each group was normalized to the wild type. Box plot as in figure 1e. Statistical test: one-way ANOVA followed by Turkey's multiple comparison test. ***p<0.001. n.s: not significant.

Scale bar =10 µm.

**Supplementary figure 7**

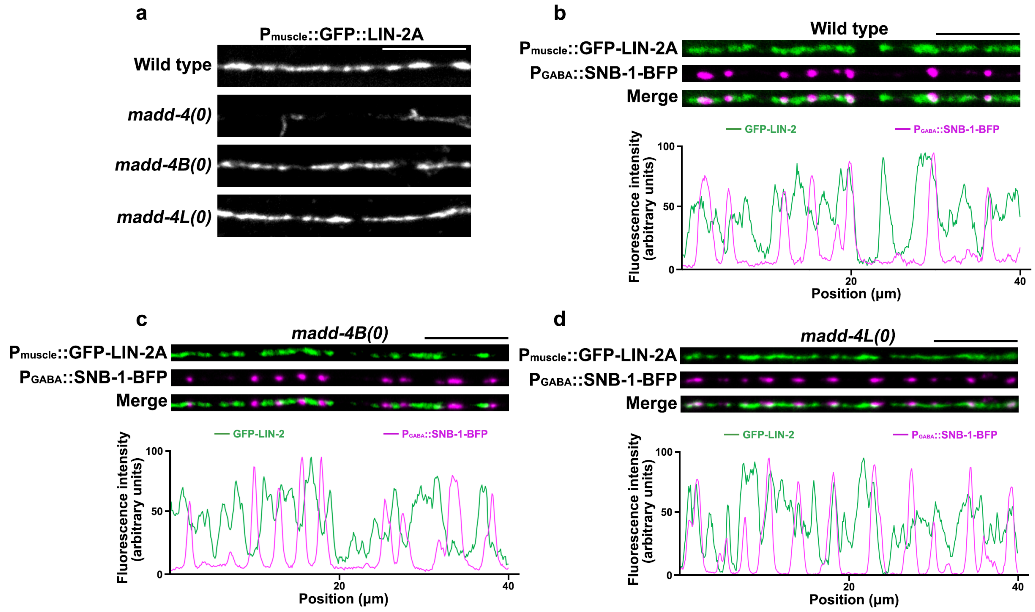


**Supplementary figure 7. MADD-4/Punctin controls the synaptic localization of LIN-2.**

(a) Confocal detection of GFP-LIN-2A expressed in muscle cells of wild-type, *madd-4(0)(kr270)*, *madd-4B(0)(tr185)* and *madd-4L(0)(ttTi103747)* mutant animals. GFP-LIN-2A fluorescence levels were quantified and examined in Figure 7G.

(b-d) The co-localization between GFP-LIN-2 expressed in muscle cells and GABAergic boutons labeled by SNB-1-BFP was assessed in the dorsal cord of wild-type animals, *madd-4B(0)(tr185*) and *madd-L(0)(ttTi103747)* mutants. The fluorescence profiles indicate GFP-LIN-2A and SNB-1-BFP fluorescence intensities along the nerve cord from the pictures above. A Mander's overlap correlation indicating the percentage of GFP-LIN-2A signal that overlaps with the SNB-1-BFP signal is presented in Figure 7H.

Scale bar = 10 µm.

**Supplementary figure 8**

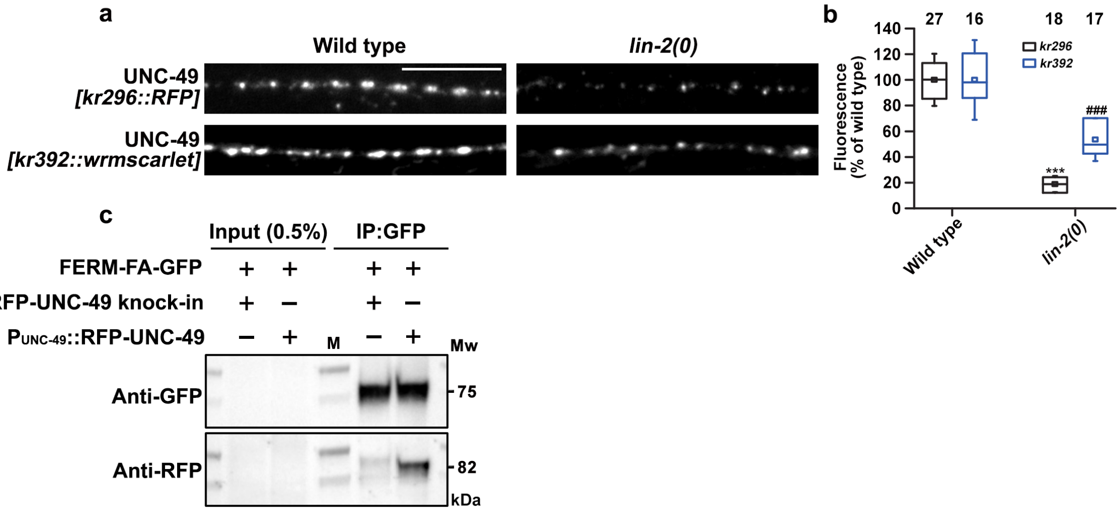


**Supplementary figure 8. Insertion of a fluorescent tag in the TM3-TM4 loop of UNC-49 increases the stability of UNC-49/GABAR clusters.**

(a-b) Confocal imaging of fluorescently-tagged $GABA_A$Rs using *unc-49(kr296::rfp)* (a N-terminal RFP knock-in in the *unc-49* locus) or *unc-49(kr392::wrmscarlet)* (a knock-in of wrmscarlet in the intracellular loop of UNC-49B in the *unc-49* locus) alleles. Fluorescence levels were quantified in wild-type or *lin-2(n397)* genetic backgrounds and normalized to the wild type. Box plot as in figure 1e. Two-way ANOVA followed by Tukey's multiple test. $^{***}$p<0.001 for *kr296*, $^{\#\#\#}$p<0.001 for *kr392*. Scale bar = 10 µm.

(c) The FERM-FA domains and the $GABA_A$R interact *in vivo*. A co-immunoprecipitation assay was performed for FERM-FA-GFP and the $GABA_A$R, expressed using the RFP-UNC-49 knock-in (*unc-49(kr296::rfp)*) or using a RFP-$GABA_A$R MosSCI (*krSi2[Punc-49::unc-49-RFP]*). 0.5% of the total *C. elegans* lysate was loaded in the input lanes, and the remaining lysate was incubated with anti-GFP nanobody TrapA beads. Immunoprecipitation of FERM-FA-GFP with anti-GFP antibodies was followed by western blot analysis with anti-GFP or anti-RFP antibody. The theoretical molecular weight of proteins is indicated to the right. M: marker. This experiment was repeated 3 times.

## Supplementary Table

Supplementary Table: MiniMos single-copy insertion alleles.

| Allele name | Construct |
|---|---|
| *krSi30* | *Pmyo-3::sfGFP::lin-2a* |
| *krSi31* | *Pmyo-3::frm-3::sfGFP* |
| *krSi32* | *Pmyo-3::frm-3(1-412aa)::sfGFP* |
| *krSi33* | *Pmyo-3::frm-3(413-1090aa)::sfGFP* |
| *krSi34* | *Phim-4::cor-1-BFP* |
| *krSi35* | *Pmyo-3::tagRFP::lin-2a* |
| *krSi39* | *Pmyo-3::frm-3(1-318aa)::sfGFP* |
| *krSi59* | *Pmyo-3::sfGFP::lin-2b* |
| *krSi60* | *Pmyo-3::sfGFP-lin-2b, line 2* |
| *krSi61* | *Pmyo-3::sfGFP-frm-3b* |

## Supplementary references


1. Kuo, Y.-C. *et al.* Structural analyses of FERM domain-mediated membrane localization of FARP1. *Scientific Reports* 8, 10477 (2018).
2. Gamblin, C. L. *et al.* Oligomerization of the FERM-FA protein Yurt controls epithelial cell polarity. *J Cell Biol* 217, 3853–3862 (2018).
3. Kosugi, S., Hasebe, M., Tomita, M. & Yanagawa, H. Systematic identification of cell cycle-dependent yeast nucleocytoplasmic shuttling proteins by prediction of composite motifs. *PNAS* 106, 10171–10176 (2009).

**Uncropped blots**

Fig 4c

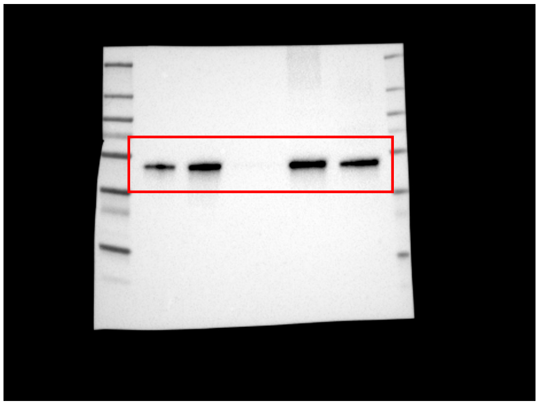

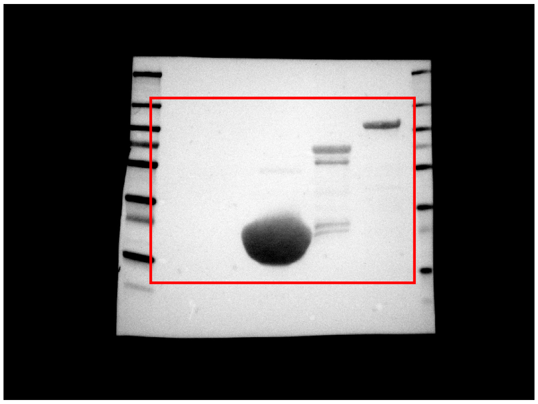

Fig 4d

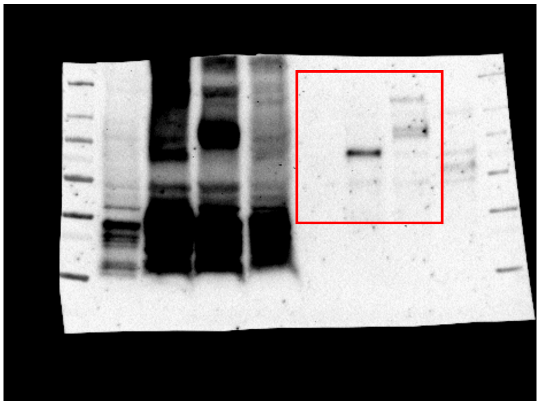

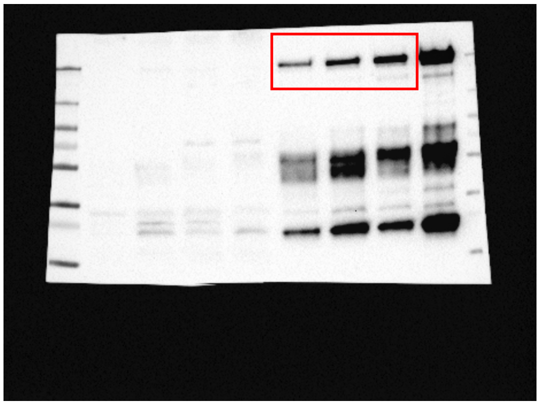

Fig 4d’

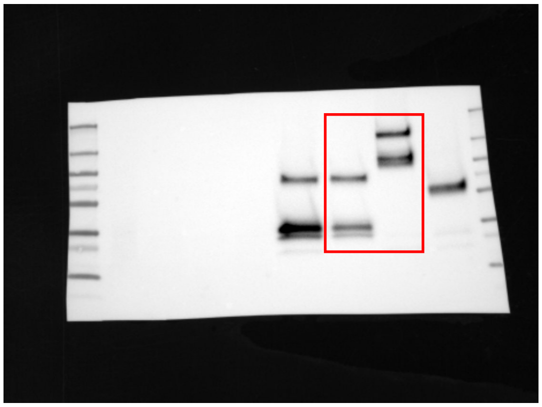

Fig 4f

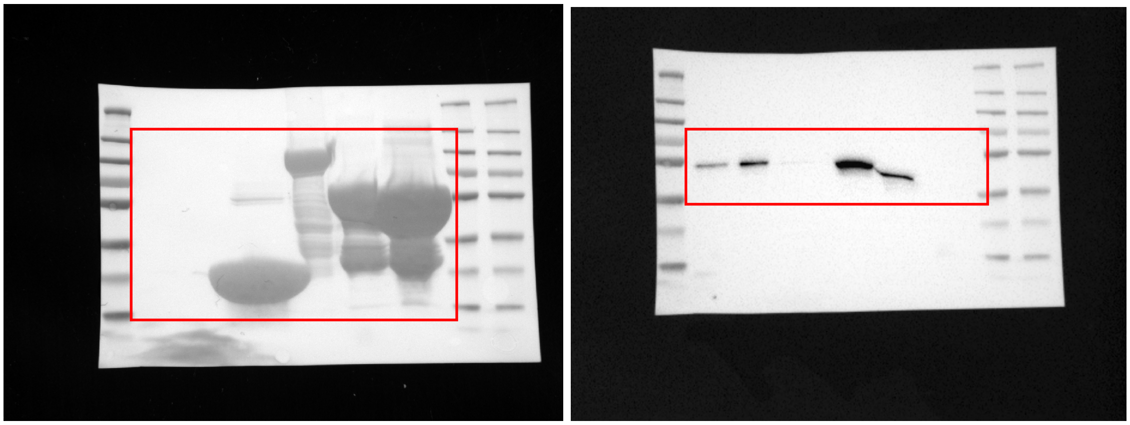

Fig 6e

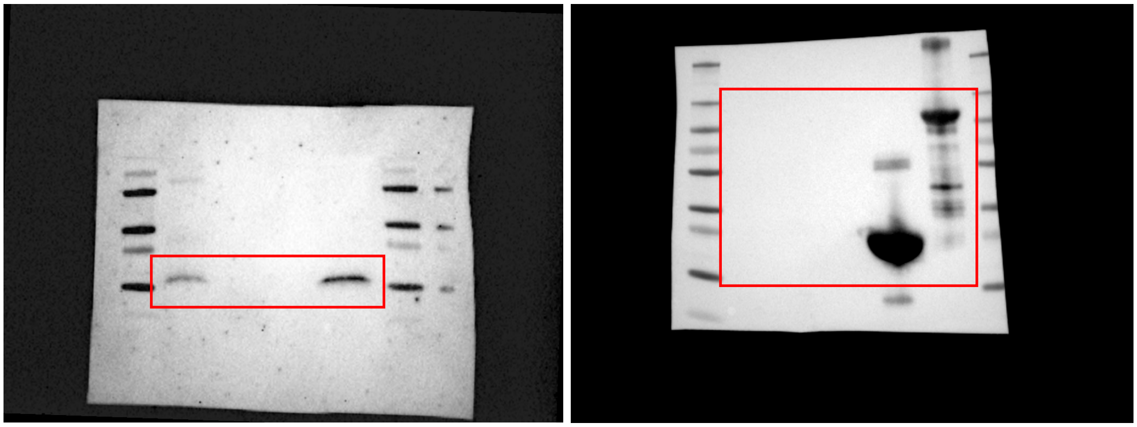

Fig 6f

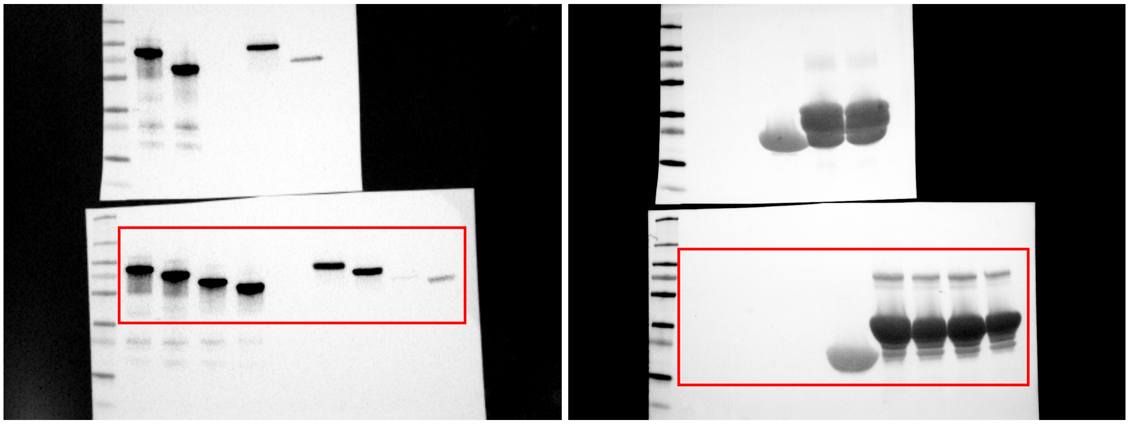

Fig 6g

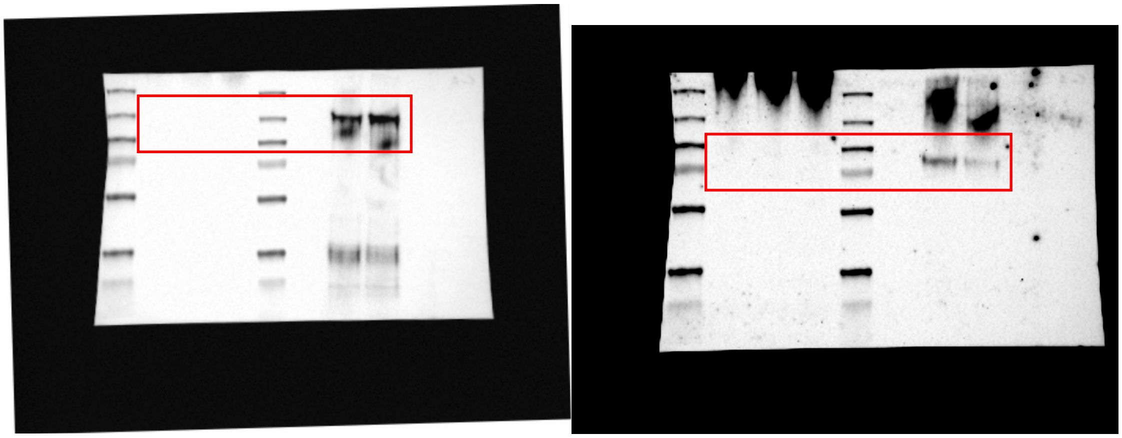

Fig 6h

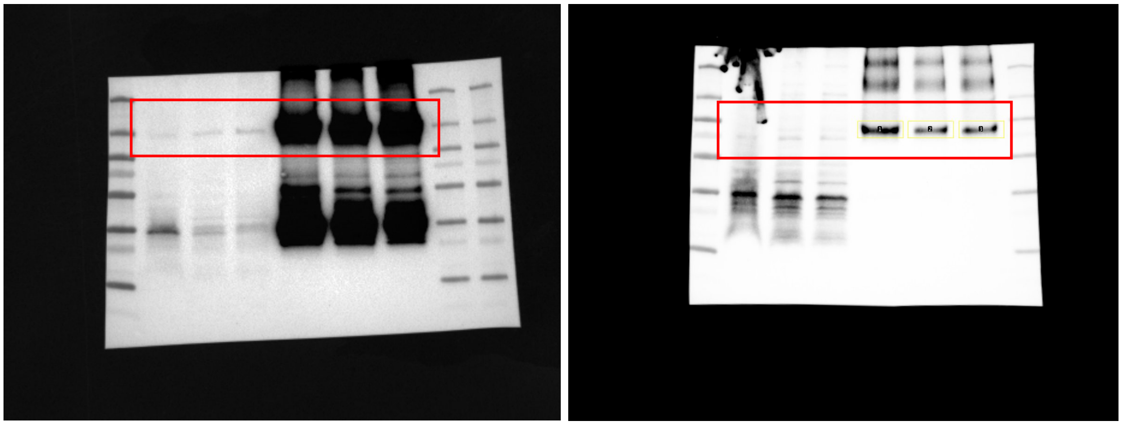

Supplement: Supplementary file 1 — Supplementary Information [file 41467_2020_16473_MOESM1_ESM.pdf]
