# Supplementary material for: The netrin receptor UNC-40/DCC assembles a postsynaptic scaffold and sets the synaptic content of GABAA receptors
Source: Nat Commun. 2020 May 29;11:2674. doi: 10.1038/s41467-020-16473-5 (PMC7260190; doi:10.1038/s41467-020-16473-5)
Supplement: Supplementary file 3 — Description of Additional Supplementary Files [file 41467_2020_16473_MOESM3_ESM.docx]

Description of Additional Supplementary Files

**Supplementary Data 1:** List of strains used in this study

**Supplementary Data 2:** List of DNA constructs generated and used in this study
